# Isolation of *Naegleria* spp. from a Brazilian Water Source

**DOI:** 10.3390/pathogens9020090

**Published:** 2020-01-31

**Authors:** Natália Karla Bellini, Ana Letícia Moreira da Fonseca, María Reyes-Batlle, Jacob Lorenzo-Morales, Odete Rocha, Otavio Henrique Thiemann

**Affiliations:** 1 Instituto de Física de São Carlos, Universidade de São Paulo, Caixa Postal 369, São Carlos, SP 13560-590, Brazil; nataliabellini@ifsc.usp.br (N.K.B.); mfanaleticia@gmail.com (A.L.M.d.F.); 2 Instituto Universitario de Enfermedades Tropicales y Salud Pública de Canarias, Universidad de La Laguna, Avda. Astrofísico Fco. Sánchez, S/N, 38203 La Laguna, Tenerife, Canary Islands, Spain; mreyesbatlle@gmail.com (M.R.-B.); jmlorenz@ull.edu.es (J.L.-M.); 3 Departamento de Ecologia e Biologia Evolutiva, Universidade Federal de São Carlos, Rodovia Washington Luis, km 235, São Carlos, SP 13565-905, Brazil; doro@ufscar.br; 4 Departamento de Genética e Evolução, Universidade Federal de São Carlos, São Carlos, SP 13565-905, Brazil

**Keywords:** *Naegleria* spp., free-living amoeba, PCR, Monjolinho River, Brazil

## Abstract

The genus *Naegleria*, of the free-living amoeba (FLA) group, has been investigated mainly due to its human health impact, resulting in deadly infections and their worldwide distribution on freshwater systems. *Naegleria fowleri*, colloquially known as the “brain-eating amoeba,” is the most studied *Naegleria* species because it causes primary amoebic meningoencephalitis (PAM) of high lethality. The assessment of FLA biodiversity is fundamental to evaluate the presence of pathogenic species and the possibility of human contamination. However, the knowledge of FLA distribution in Brazil is unknown, and to rectify this situation, we present research on identifying *Naegleria* spp. in the Monjolinho River as a model study. The river is a public Brazilian freshwater source that crosses the city of São Carlos, in São Paulo state, Brazil. Five distinct sampling sites were examined through limnological features, trophozoites culturing, and PCR against internal transcribed spacer (ITS) regions and 5.8S rRNA sequences. The results identified *N. philippinensis, N. canariensisi, N. australiensis, N. gruberi, N. dobsoni* sequences, as well as a *Hartmannella* sequence. The methodology delineated here represents the first Brazilian *Naegleria* spp. study on a freshwater system. Our results stress the urgency of a large scale evaluation of the presence of free-living amoebas in Brazil.

## 1. Introduction

*Naegleria* is a genus that comprises single-celled, heterotrophic protists that are widely distributed in natural environments [1,2,3]. The 47 species identified up to now exist as free-living amoebas (FLAs) with a bacteria-based feeding habit and binary fission division [4]. In unfavorable environmental conditions, they transform from trophozoites to the cyst stage as a resting form [5]. Additionally, a third stage, commonly biflagellate, can be formed to seek a better surrounding [6]. The known exceptions are *Naegleria indonesiensis, N. chilensis, N. paradobsoni* and *N. neochilensis* for which a flagellate form has never been identified [4].

*Naegleria* is described as an amphizoic genus, as four species are able to thrive not only as free living organisms but also as parasites: *Naegleria fowleri, Naegleria australiensis, Naegleria philippinensis* and *Naegleria italica* [7,8,9]. Since *N. fowleri* withstands higher temperatures, as found in geothermal sources and heated recreational aquatic systems, the species has been classified as thermophilic [3,10] with the ability to grow in temperatures ranging from 30 to 46 ℃ [11,12]. Other species, like *N. australiensis*, *N. italica*, *N. lovaniensis* and *N. philippinensis*, have been recognized as thermo tolerant as well [4,13]. 

*Naegleria* is phylogenetically grouped with another amphizoic genus, *Vahlkampfia*, within the Vahlkampfiidae family, the clade Discoba, and the super group Excavata [5,14,15,16]. In addition, the *Acanthamoeba, Balamuthia, Sappinia* and *Vermamoeba* genera are pathogenic FLA groups whose main threats to humans are granulomatous amoebic encephalitis (GAE) and amoebic keratitis (AK) [17,18,19]. GAE is caused by *Acanthamoeba* spp., *Balamuthia mandrillaris and Sappinia pedata,* with several cases in immunocompromised persons, whereas AK is caused mainly by *Acanthamoeba* spp. with prevalence in contact lens wearers [19,20,21]. More recently, *Vermamoeba vermiforms,* a common free-living amoebae, was identified in one corneal scraping from an AK case [22].

Primary amoebic meningoencephalitis (PAM) is a fatal infection caused by *Naegleria fowleri,* whose trophozoite can reach the central nervous system (CNS) through the olfactory neuroepithelial pathway [23]. In 95% of the reported cases, death results from seven to fourteen days after the appearance of symptoms [18,24,25,26,27]. This alarmingly low survival rate has been correlated with the difficulty in diagnosis and the low efficacy of the available therapy, commonly based on antifungal and antibacterial drugs [28,29]. Additionally *N. australiensis, N. philippinensis* and *N. italica* are potentially pathogenic species because they have presented infectivity in mice models [7,8,9]. Considering that the amoeba infects the host by direct contact and that most PAM patients have a history of water contact (e.g., bathing in freshwater) [23] prior the outcome of encephalitis, the investigation on the environmental distribution of these species is of great public health importance to prevent new cases and is critical to deepen the knowledge on *Naegleria* diversity.

South American countries have faced a scarcity of information on *Naegleria* distribution throughout the world [25]. In Brazil, the first occurrence of *N. fowleri* was reported in an artificial lake from the city of Rio de Janeiro [30]. Later, the presence of *Naegleria* genus was linked to dust and biofilm from hospitals in the cities of Presidente Prudente [31] and Porto Alegre [32]. The cyst capability of resisting to desiccation enables PAM infections via the dry infection route [33]. In 2009, *N. fowleri* was correlated to dust in two campuses of a university in the city of Santos [34]. Regarding Brazilian PAM cases, a 1985 report diagnosed the infection through an immunological analysis of the cerebral tissue of a deceased patient [30], and two reports identified *N. fowleri* infection in cattle, based on histological and immunohistochemical analysis [35,36]. These reports lacked molecular techniques to confirm the findings as a complementary strategy to combine with the morphological and physiological characterization of the isolated organism. Currently, the ribosomal RNA gene, particularly the region of the internal transcribed spacers (ITS1 and ITS2), and the 5.8S ribosomal gene have been used in the newest studies of *Naegleria* [37,38,39,40,41]. 

Accentuating the lack of reports, Brazil is one of the most promising countries for *Naegleria* dispersion because it harbors about twenty percent of the global freshwater [42], the most prevalent habitat in which *Naegleria* has been found. Taking into account this fact when considering the biodiversity of *Naegleria* species, pathogenic or non pathogenic, in Brazilian freshwater courses, we undertook this initiative by investigating a river in the city of São Carlos, São Paulo state, as a model system. The Monjolinho River belongs to a basin that covers São Carlos city and neighboring districts in the state of São Paulo, and it reaches a watershed dimension of 275 km^2^. The area belonging to São Carlos city is under intense anthropogenic activity and is highly impacted with domestic and industrial sewage, in addition to agricultural runoff [43,44]. Therefore, the present research examined five distinct sampling sites in which *Naegleria* spp. were isolated and characterized by the PCR amplification and DNA sequencing of the ITS1, 5.8S, and ITS2 regions of the total DNA from each site. This characterization, when combined with the morphological investigation after culturing and the limnological characterization of the water, allowed us to identify the presence of five *Naegleria* spp. in a Brazilian freshwater course.

## 2. Results

### 2.1. Limnologic Characterization

**Figure 1:**
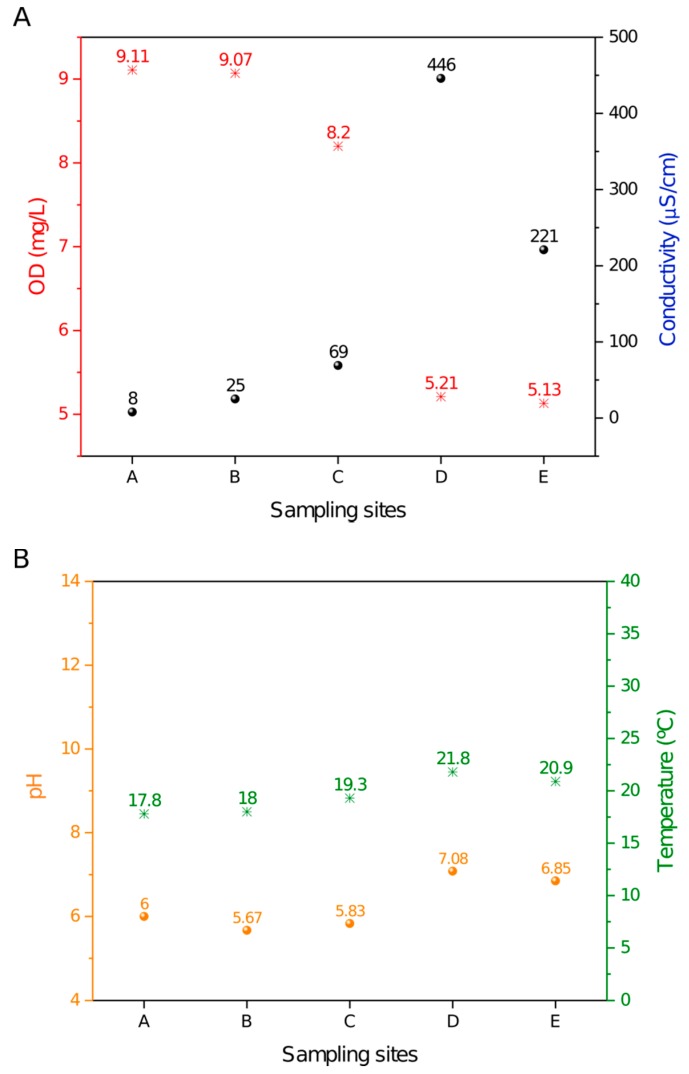
Limnological profile throughout the sampling sites in the Monjolinho River. (**A**) DO (dissolved oxygen) and conductivity values plotted along the river samples. (**B**) Temperature and pH values plotted along the river samples.

The DO (dissolved oxygen), conductivity, pH and temperature measurements that were acquired in situ along the Monjolinho River are summarized in Figure 1.

Samples A–E along the river displayed a decrease in the dissolved oxygen that was concomitant with the increase in the conductivity values. The D position was striking, as it showed an abrupt accentuation in both data (Figure 1A). This result correlated well with site D being located at a domestic sewage discharge area where organic compounds are dissolved in the water, leading to a high ionic concentration that is consistent with a decrease in DO. The temperature and pH values were relatively constant from sites A to E (Figure 1B) and apparently were not influenced by the domestic sewage discharge. A small increase in temperature was observed, most likely due to daily variations in ambient temperature. As shown in Figure 1B, the recorded pH values ranged from 5.67 to 7.08, and temperatures ranged from 17.8 to 21.8 °C. Thus, pH and temperature exhibited a smaller variation among sites as compared to the DO and conductivity values along the five sampling sites.

### 2.2. Light Microscopy

**Figure 2:**
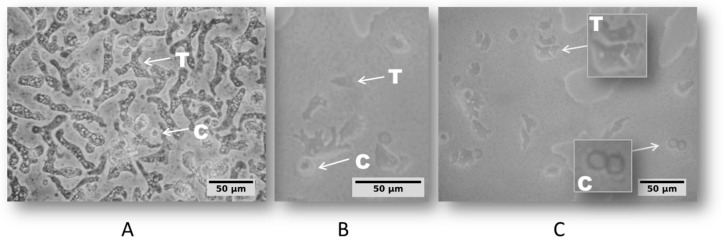
Light microscopy images of amoeba cells on non-nutrient agar (NNA) plates from sampling site D that were cultivated at 26 °C (**A**), 37 °C (**B**), and 44 °C (**C**). Arrows indicate trophozoite (T) and cyst (C) cells that were observed with an inverted microscope (Nikon TS 100) at ×400 magnification.

The daily microscopic examination of non-nutrient agar (NNA) plates allowed for the identification of amoeba growth in 100% of the sampling sites that were covered in this study. According the amoeba cell morphologic profile defined by Page’S Key [45], two life cycle stages, trophozoite and cyst, were observed (Figure 2).

The appearance of trophozoites and cystic forms was regularly inspected until the cultures reached 14 days outgrowth and the correspondence between culture findings (Figure 2) and their respective species was addressed by the molecular approach, detailed next.

### 2.3. PCR Amplification and Sequence Analysis 

**Figure 3:**
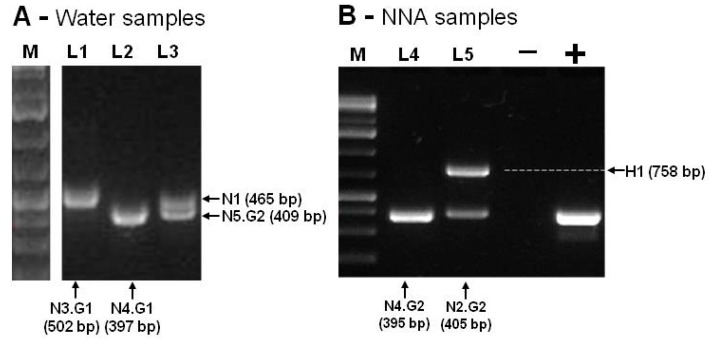
PCR amplification of *Naegleria* internal transcribed spacer (ITS) rDNA region. In (**A**), the lanes L1 and L2 correspond to the isolates of sampling site C, and L3 corresponds to the isolates of sampling site E. In (**B**), the lanes L4 and L5 correspond to sampling site D cultivated at 37 and 44 °C, respectively. The arrows indicate the amplified products that correspond to *Naegleria* ITS genes (N1–N5) and the *Hartmannella* (H1) gene. The molecular marker (M, Gene Ruler 1 kb Plus DNA Ladder, Thermo Fisher Scientific), positive (+) control, and negative (-) control are also displayed.

By using the Ng.spp_FW and Ng.spp_RV primers, the PCR products obtained ranged from 395bp to 502bp, corresponding to the expected size of *Naegleria* spp amplicon (N1 to N5). An amplicon of about 750bp was obtained in one sample, consistent with the expected fragment size of *Hartmannella* spp (H1), as shown in Figure 3.

Figure 3 summarizes the amplification results that were obtained by using Ng.spp_FW and Ng.spp_RV primers. The negative control was performed with DNA-free water, and the positive control was performed with DNA of *Naegleria gruberi* ATCC (American Type Culture Collection) 30224. All these amplicons (Figure 3A,B) were isolated from the agarose gel, sequenced, and compared with each corresponding species through BLASTn searches. Table 1 summarizes the results for all the sampling sites. 

Our findings were 95%–100% identical with GenBank *Naegleria* spp. reference sequences, with a query coverage of 98%–100% at BLASTn alignment, and the variants “.G1” and “.G2” mean that differences were detected on the nucleotide composition within the same species. The number of substitutions per site from averaging over all sequence pairs of the *Naegleria* reference sequences was 0.06, with a standard error estimate of 0.01, revealing a high sequence conservations among *Naegleria* species. However, this internal variability on the rDNA sequences that were detected within the *Naegleria* species DNA was not significant enough to suggest the identification of new species.

The sequencing results from the NNA plate samples (Table 1) revealed the presence of four distinct *Naegleria* species, *N. philippinensis, N. canariensis*, *N. australiensis* and *N. gruberi*, as well as one *Hartmannella* species. According to the D site findings, *N. australiensis* was capable of growing at 44 ℃. Additionally, *N. dobsoni* could be isolated when taking into account the identifications directly from water (Table 1).

### 2.4. Phylogeny

**Figure 4:**
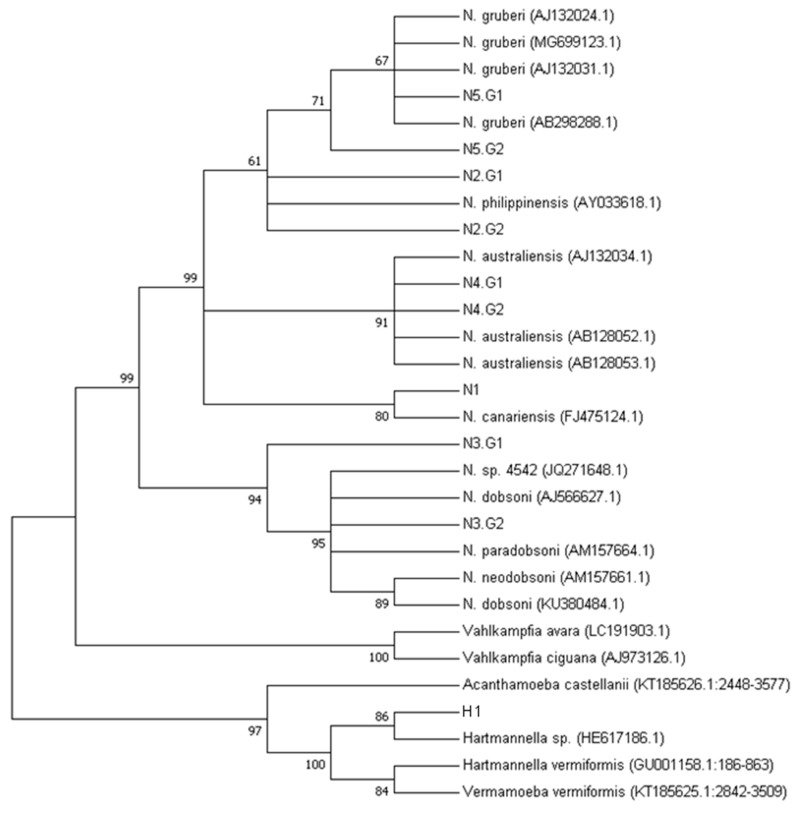
Evolutionary relationship among *Naegleria* spp., *Hartmannella* spp. and *Vahlkampfia* spp. based on internal transcribed sequences (ITS1 and ITS2 regions) and 5.8S sequence. The bootstrap consensus tree was inferred from 1000 replicates. Branches corresponding to partitions reproduced in less than 50% of the bootstrap replicates are collapsed. *Hartmannella vermiformis* and *Acanthamoeba pearcei* were used as outgroups for the *Vahlkampfia* and *Naegleria* clades.

The neighbor joining (NJ) tree of the aligned ITS and 5.8S rDNA sequences (Figure S1) clearly show the N5 (G1 and G2 variants) branching with the *Naegleria gruberi* species. The N4 sequence belonged to the *N. australiensis* group, N2 belonged to *N. philippinensis*, N1 belonged to *N. canariensis,* and N3 belonged to the *N. dobsoni* clades. The sequence resulting from the 758bp amplicon (H1) was identified as belonging to the *Hartmannella* and *Acanthamoeba* clades (Figure 4) with good bootstrap support.

## 3. Discussion

### 3.1. Eutrophication along the Monjolinho River 

The samples were collected along the Monjolinho river, which passes through the city of São Carlos in the São Paulo state, Brazil. Five sampling sites were selected. Site A represented the river headwaters and the catchment area [43]. The subsequent sampling sites were located within (B and C) and nearby (D) the urban area where the population can use the water for agricultural, domestic or recreational purposes. According the limnological measurements shown in Figure 2, the dissolved oxygen and conductivity results revealed the degree of eutrophication of the Monjolinho River basin, as these are the known parameters that are to evaluate levels of organic material contaminating the water [46]. From the headwater towards the two subsequent sampling sites, a gradual increase in conductivity values is observed, together with a decrease of dissolved oxygen. However, the severe reduction of the electrical conductivity of sites D to E was likely due to the sewage treatment plant located between sites D and E. Increment of the electrical conductivity of water are closely related to organic matter decomposition, anthropogenic activities, and soil flushing, as these activities elevate potassium, magnesium, calcium, carbonate and sulphate concentrations [47]. The lowest DO values that were found in the D and E sampling sites were below the recommendations from the Brazilian Council of Environmental Regulations, which has determined that DO values should not be lower than 6.0 mg.L-1 as an environmental parameter of water quality [48]. The slight increase of the temperature from headwater to the last meander was likely due to the daily temperature change once the sampling followed the longitudinal course of the river (A–E), and it took an interval of five hours from the first to the last site. The gentle increase in the pH value was probably a consequence of the presence of alkaline compounds, such as carbonates and calcium, that could increase the pH and were also responsible for the increase of the electrical conductivity. Additionally, detergents that are commonly encountered in domestic wastewater have the potential to increase the pH to levels that were consistent with the recorded values compared to the upstream sampling sites [49]. Nevertheless, all physical and chemical features registered in the Monjolinho River could harbor *Naegleria,* as described in this paper.

### 3.2. Presence of Naegleria Thermophilic Species in Brazil 

Our cultivation results, particularly the trophozoite growth observed at 44 °C, indicated the presence of species with pathogenic potential, as it has been discussed in earlier studies that all pathogenic species of *Naegleria* have the capability of tolerating temperature of up to 40 °C [16]. The observed capability of *N. australiensis* to grow and sustain its growth at 44 °C broadens the thermotolerance that has thus far been associated with this species, because no earlier studies have demonstrated its tolerance to 44 °C, either from environmental samples or in laboratorial conditions [4]. To date, the highest temperatures tolerated by *N. australiensis* have been reported to be in the range of 40–43 °C [2,11,37,50,51]. On the other hand, *N. philippinensis* has been reported to grow at 40 °C [4], which is consistent with our results, which confirmed its inability to withstand a temperature of 44 °C. 

The identification of *N. australiensis* and *N. philippinensis* (Table 1) brings an important issue to be explored due to their capability to cause encephalitis in experimental animals [52,53], and their presence in the river water represents a threat not only to the ichthyofauna but also to domestic mammals that use the river as water supply. Moreover, just because the causative agent of PAM in humans, *N. fowleri*, was not recovered in this collection does not mean that there is no need for the frequent evaluation of this and other river streams. These assessment are becoming urgent with the rising temperatures that are being experienced due to global warming effects [11,29,54].

### 3.3. Naegleria spp. Diversity in Brazil

Besides the identification of *N. australiensis* and *N. philippinensis*, this study also identified *N. canariensis*, *N. gruberi,* and a *Harmannella* spp. through the same morphological approach combined with sequencing analysis, as shown by the bootstrap supported phylogenetic tree (Figure 4), although the short length of the ITS-5.8S sequences did not resolve the polytomy within each species. The potential of the selected primers to amplify two distinct free-living amoeba genera has been recently reported in two studies from the Nile River, Egypt [38] and from regions of Malaysia [39]. By analyzing the DNA sequences that were directly extracted from the river water (Table 1), one additional *Naegleria* species could be identified: *N. dobsoni*. Similarly, some species were not isolated in the direct approach, but, after enriching the trophozoites presence by culturing, we could obtain them. Thus, we highlight the importance on performing both complementary PCR analyses when using the DNA extracted directly from the river and the DNA after culturing. 

To the best of our knowledge, this is the first report of *N. philippineneis*, *N. australiensis*, *N dobsoni* and *N. gruberi* in South America environmental samples. To date, reports of the presence of *Naegleria* from South American countries have relied on the identification of *N. fowleri* from suspected PAM victims. For instance, two Venezuelan [55] and five Brazilian [30] PAM cases have been registered. One additional *Naegleria* species has been identified in the public swimming pools of Santiago, Chile [56]. Thus, our findings represent a significant improvement on our knowledge on the presence and diversity of *Naegleria* in South America.

## 4. Materials and Methods 

### 4.1. Description of the Geographical Area and Sample Collection

**Figure 5:**
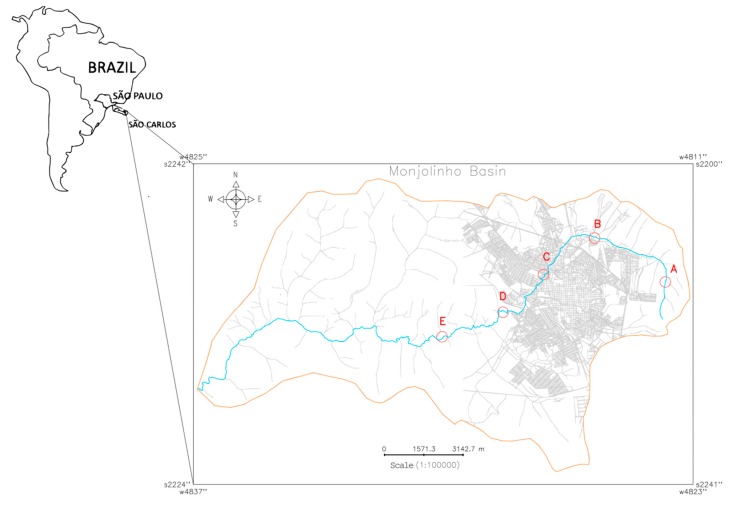
Cadastral map of the Monjolinho River Basin, São Carlos, São Paulo (Brazil) that shows the Monjolinho river (blue line) and sampling sites A–E (in red letters and circles) that were investigated in this study.

Five water samples (A–E) were collected from the Monjolinho river, which runs through the city of São Carlos, São Paulo, Brazil. Different levels of human intervention along the Monjolinho River basin were considered when selecting the five sampling sites that are described in the present study. To insert each sampling site (Universal Transverse Mercator, UTM) coordinates in the Monjolinho River basin image, a geographic information system (GIS) named the Georeferenced Information Processing System (SPRING 5.5.5, http://www.dpi.inpe.br/spring/english/index.html) was used. The resulting cadastral map (Figure 5) revealed the basin limit and the respective geographic positions of each sampling site along the main stream of the Monjolinho as follows: headwater (A: 22°0’2.87” S, 47°50’8.96” W), peri-urban stream (B: 21°58’40.6884” S, 47°52’25.50” W), urban stream (C: 21°59’45.40” S, 47°54’6.32” W), domestic sewage discharge (D: 22° 0’ 52.41” S, 47° 55’ 26.65” W), and after a sewage treatment plant (E: 22°1’34.92” S, 47°57’26.29” W). The samples were collected in October, 2017 in the dry season by using one liter sterile glass flasks in a range of 10–15 cm of the water surface, following a literature recommendation [57].

### 4.2. Limnology and Sampling Process

The global positioning system (GPS) coordinates of each sampling site were registered. The limnological data of the sampling sites, such as dissolved oxygen (DO), hydrogen ionic potential (pH), and temperature, were recorded by a portable water quality analyzer (Horiba U-10). Within 12 hours after collection, the samples were homogenized and filtered through a granulometric sieve (BERTEL-MESH 120, pore diameter of 0.125 mm). Five hundred milliliters of the sieved samples were used for culturing, and the remaining 500 mL were used for the PCR amplification of the target DNA and the DNA sequencing. 

### 4.3. Amoeba Isolation and Culturing

The water samples were filtered in a 0.45 µm cellulose nitrate membrane (Nalgene^®^) and cut in nine pieces, and each piece was placed in a 2% agar NNA plate [58] for each sampling site. They were then overlaid with heat-inactivated *Escherichia coli* (60 °C for 30 min) [53]. Three of each sealed plates were incubated at 26, 37, and 44 °C [39,40]. 

### 4.4. Morphological Characterization

The cultures were examined daily for the presence of amoeba growth and the trophozoites identified were sub-cultured onto new NNA plates and their growth was inspected in an inverted Nikon Eclipse TS 100 microscope. To infer the taxonomic identity based on the Page′s classification key, the photomicrographs were screened for amoeba-related morphological characters [45]. The trophozoites were harvested in 2 mL Page′s saline solution (PAS) [58], centrifuged (800× *g*, 10 min), suspended in a freezing solution (9:1, heat inactivated fetal bovine serum:DMSO), and then stored in liquid nitrogen vapor.

### 4.5. DNA Extraction, Amplification and Sequencing

To perform the molecular identification directly from the water samples, 500 mL of filtered water was centrifuged at 3000× *g* for 15 min at room temperature and washed once in phosphate-buffered saline (PBS). The final pellet was used for DNA extraction. Likewise, the NNA-cultured trophozoites were washed once with 5 mL PAS on the agar surface to homogenization in a plate shaker (60 rpm, 10 min). The suspended cells were poured into a canonical 15 ml tube and centrifuged at 1000× *g* for 10 min at RT, and the pellet was solubilized with 50 µL of ultrapure water (Millipore, Burlington, EUA) prior to DNA extraction. The commercial extraction kit DNeasyPowerSoil^®^ Kit (QIAGEN, Hilden, Germany) was used to achieve high DNA purity and yield for both input materials—the water samples and the NNA-cultured trophozoites. The DNA concentration was quantified in a Nanodrop 2000 Spectrophotometer (Thermo Scientific, Waltham, MA, USA), analyzed by agarose-gel electrophoresis, and amplified by the polymerase chain reaction (PCR).

The DNA was amplified with *Naegleria* genus-specific primers, Ng.spp_FW 5′-GAACCTGCGTAGGGATCATTT-3′ and Ng.spp_RV 5′-TTTCTTTTCCTCCCCTTATTA-3′, to the internal transcribed spacer regions (ITS1 and 2) that comprise the 5.8S rDNA gene that was previously adopted in phylogenetic studies [37,38,39,40,41]. The PCR reactions contained Taq High Fidelity Pol Master Mix 2x (Red, Cellco Biotec, São Carlos, Brazil), 100 ng of the DNA template, and 200 nmoles of each primer. The reactions were incubated in a T100 Thermal Cycler (BIO-RAD, Hercules, CA, USA) under a cycling condition of 95 °C for 3 min followed by 35 cycles at 95 °C for 30 s, 50 °C for 30 s, 72 °C for 1 min, and a final extension at 72 °C for 2 min. Amplified fragments were examined in SYBR Safe-stained 2% agarose gels. The amplified DNA bands were purified by using the Agarose Gel Extraction Kit (Cellco Biotec), cloned into a pJET 1.2/blunt cloning vector (Thermo-Scientific) and transformed into *E. coli* TOP 10 competent cells. The plasmid DNA was isolated with Fast-n-Easy Plasmid Mini-Prep Kit (Cellco Biotec) and sequenced in triplicate in both directions in a 3130 Genetic Analyzer (Thermo Scientific); therefore, each base was independently sequenced six times. The chromatograms were carefully examined with DNA analysis software (SnapGene Viewer), and the nucleotide homology was investigated through Basic Local Alignment Search Tool (BLAST) at the National Centre for Biotechnology Information (NCBI) blasting with Eukarya Domain sequences.

### 4.6. Phylogenetic Inferences 

The phylogenetic analysis was based on the ITS and 5.8 S sequences from this work. The consensus sequences were aligned with the *Naegleria* rDNA reference sequences that were already deposited in the GenBank database by using ClustalW software with the following gap penalties: 20/0.1 and 15/6.66 to gap opening/extension for the pairwise and multiple alignments, respectively. The final alignment was used for the phylogenetic analysis with Molecular Evolutionary Genetics Analysis (MEGA7) to build a phylogenetic tree based on neighbor joining (NJ) inference method with the Tamura–Nei statistic model [59]. In order to determine the statistical reliability of each node, 1000 bootstrap replicates were performed.

## 5. Conclusions

In this study, we describe the identification of *Naegleria* along the Monjolinho River Basin with morphological and molecular approaches. Although not exhaustive, the combination of both morphological and molecular methodologies allowed for a more complete understanding of the amoebas that were present. Because earlier Brazilian studies focused the *Naegleria* environmental investigations based on morphological analyses, they reported their information at genera level. In this context, the present research was the first Brazilian study to perform a molecular approach to detect *Naegleria* in an environmental sample and in which the findings could change the scenario by revealing the presence of *Naegleria* at the species level. Thus, this study increases knowledge on diversity due the identification of five distinct *Naegleria* species and one *Harmannella* gene. The limnological characterization that was accomplished by this study allowed for the assessment of eutrophication, as the D sampling site was found to be the most eutrophic compared to the remaining four sites. Additionally, this site was the most diverse regarding the species that were, identified as it had three of the five *Naegleria* spp. and the *Hartmannella* species that were found in the river. Although *N. fowleri* was not recovered in this survey, the presence of *N. australiensis* and *N. philippinensis,* distributed from the headwater to the fourth sampling site, indicates that potentially pathogenic species could grow in the Monjolinho River. Even if they have never been reported in human infections, their potential to cause encephalitis in laboratory conditions renders these species worthy of a more careful investigation. Since both species were found in the most eutrophic sampling site, there is a need to investigate the surrounding freshwater systems with similar limnological conditions that could favor their presence and dispersion. Our findings strengthen the importance of combining morphological approaches with molecular investigations in order to reach a deeper investigation of *Naegleria* spp. in environmental samples. This study highlights the need to perform further investigations that address the presence and distribution of potentially pathogenic FLA genera in Brazil.

## Figures and Tables

**Table 1:** *Naegleria* and *Hartmannella* isolates from NNA cultures (1) and directly from the water (2) through sampling sites of the Monjolinho River (A–E) in São Carlos, São Paulo state.

Sample Sites	Culture Temperature	1-Isolates from NNA Cultures		2-Isolates Directly from Water Samples	
		Code	BLASTn/Accession ^1^	Code	BLASTn/Accession ^1^
A	26 ℃	N2.G2	*N. philippinensis*/*LC191904.1*	N1	*N. canariensis*/*FJ475124.1*
	37 ℃	N1	*N. canariensis*/*FJ475124.1*		
B	26 ℃	N1	*N. canariensis/FJ475124.1*	N2.G1 N1	*N. philippinensis;* *N. canariensis/FJ475124.1*
	
C	26 ℃	N1	*N. canariensis/FJ475124.1*	N4.G1 N3.G1 N3.G2	*N. australiensis*/AB128053.1; *N. dobsoni/KU380484.1* *N. dobsoni/KU380484.1*
	37 ℃	N1	*N. canariensis/FJ475124.1*		
D	26 ℃	N4.G2	*N. australiensis/AB128052.1*	N2.G1 N5.G1	*N. philippinensis*/AY033618.1; *N. gruberi*/MG699123.1
	37 ℃	N2.G2	*N. philippinensis/LC191904.1*		
		H1	*Hartmannella*/HE617186.1		
	44 ℃	N4.G1	*N. australiensis*/AB128053.1		
E	26 ℃	N5.G2	*N. gruberi*/MG699123.1	N5.G2 N1	*N. gruberi*/MG699123.1; *N. canariensis/FJ475124.1*
	37 ℃	N1	*N. canariensis/FJ475124.1*		

^1^ Gene-Bank accession numbers.

## Supplementary Materials

The following are available online at https://www.mdpi.com/2076-0817/9/2/90/s1, Figure S1: Aligned ITS and 5.8S rDNA sequences.
