# Supplementary material for: Isolation of Naegleria spp. from a Brazilian Water Source
Source: Pathogens. 2020 Jan 31;9(2):90. doi: 10.3390/pathogens9020090 (PMC7169387; doi:10.3390/pathogens9020090)
Supplement: Supplementary file 1 [file pathogens-09-00090-s001.pdf]

Supplementary figure

Multiple sequence alignment of the ITS and 5.8S rDNA sequences, excluding sites with alignment gaps. Yellow highlighting displays the conserved nucleotides among the sequences. The ITS1, 5.8S, ITS2 and LSU markings were based on the *Naegleria* genes alignment to delimited regions.

ITS1 | 5.8S

Domain: Data		
N. australiensis (AJ132034.1)	GAAACCTTTTTTCCCCACTGTGCAATGGAGCACACGGCTT	[416]
N. gruberi (AJ132024.1)	GAAACCTTTTTTCCCCACTGTGCAATGGAGCACACGGCTT	[416]
N. gruberi (AJ132031.1)	GAAACCTTTTTTCCCCACTGTGCAATGGAGCACACGGCTT	[416]
N. philippinensis (AY033618.1)	GAAACCTTTTTTCCCCACTGTGCAATGGAGCACACGGCTT	[416]
N. dobsoni (AJ566627.1)	GAAACCTTTTCCATCCACTGTGCAATGGAGCACACGGCTC	[416]
N. paradobsoni (AM157664.1)	GAAACCTTTTCCATCCACTGTGCAATGGAGCACACGGCTC	[416]
N1	GAAACCTTTTTTCCCCACTGTGCAATGGAGCACACGGCTT	[416]
N2.G1	GAAACCTTTTTTCCCCACTGTGCAATGGAGCACACGGCTT	[416]
N2.G2	GAAACCTTTTTTCCCCACTGTGCAATGGAGCACACGGCTT	[416]
N3.G1	GAAACCTTTTTACCCCACTGTGCAATGGAGCACACGGCTC	[416]
N3.G2	GAAACCTTTTCCATCCACTGTGCAATGGAGCACACGGCTC	[416]
N4.G1	GAAACCTTTTTTCCCCACTGTGCAATGGAGCACACGGCTT	[416]
N4.G2	GAAACCTTTTTTCCCCACTGTGCAATGGAGCACACGGCTT	[416]
N5.G1	GAAACCTTTTTTCCCCACTGTGCAATGGAGCACACGGCTT	[416]
N5.G2	GAAACCTTTTTTCCCCACTGTGCAATGGAGCACACGGCTT	[416]
H1	CCTTCGAGAACGAACCTTTCGACGACGGATACCTCGGTTC	[416]
N. canariensis (FJ475124.1)	GAAACCTTTTTTCCCCACTGTGCAATGGAGCACACGGCTT	[416]
N. neodobsoni (AM157661.1)	GAAACGAATTCCATCCACTGTGCAATGGAGCACACGGCTC	[416]
Hartmannella vermiformis (GU001158.1:186-863)	CCTTCGAGAACGAACCTTTCGACGACGGATACCTCGGTTC	[416]
N. gruberi (AB298288.1)	GAAACCTTTTTTCCCCACTGTGCAATGGAGCACACGGCTT	[416]
N. gruberi (MG699123.1)	GAAACCTTTTTTCCCCACTGTGCAATGGAGCACACGGCTT	[416]
N. dobsoni (KU380484.1)	TGGACCTTTTCCATCCACTGTGCAATGGAGCACACGGCTC	[416]
N. sp. 4542 (JQ271648.1)	GAAACCTTTTCCATCCACTGTGCAATGGAGCACACGGCTC	[416]
N. australiensis (AB128052.1)	GAAACCTTTTTTCCCCACTGTGCAATGGAGCACACGGCTT	[416]
N. australiensis (AB128053.1)	GAAACCTTTTTTCCCCACTGTGCAATGGAGCACACGGCTT	[416]
Acanthamoeba castellanii (KT185626.1:2448-357	ATATCGTATTTTAATCACCTAACAACGGATATCTTGGTTC	[416]
Vermamoeba vermiformis (KT185625.1:2842-350	CCTTCGAGAACGAACCTTTCGACGACGGATACCTCGGTTC	[416]
Vahlkampfia avara (LC191903.1)	GCGTTCGTGAGTAACCACTTTGCGATGGATCACTTGGCTA	[416]
Vahlkampfia ciguana (AJ973126.1)	ATGTTCGAAAATAACCACTTTGCGATGGATCACTTGGCTA	[416]
Hartmannella sp. (HE617186.1)	CCTTCGAGAACGAACCTTTCGACGACGGATACCTCGGTTC	[416]

N. australiensis (AJ132034.1)	ATGCATCGATGAAGCCCGCGGCAAAAAGCGATATGTAATG	[456]
N. gruberi (AJ132024.1)	ATGCATCGATGAAGCCCGCGGCAAAAAGCGATATGTAATG	[456]
N. gruberi (AJ132031.1)	ATGCATCGATGAAGCCCGCGGCAAAAAGCGATATGTAATG	[456]
N. philippinensis (AY033618.1)	ATGCATCGATGAAGCCCGCGGCAAAAAGCGATATGTAATG	[456]
N. dobsoni (AJ566627.1)	GTGCATCGATGAAGCCCGCGGCAAAAAGCGATATGTAATG	[456]
N. paradobsoni (AM157664.1)	GTGCATCGATGAAGCCCGCGGCAAAAAGCGATATGTAATG	[456]
N1	ATGCATCGATGAAGCCCGCGGCAAAAAGCGATATGTAATG	[456]
N2.G1	ATGCATCGATGAAGCCCGCGGCAAAAAGCGATATGTAATG	[456]
N2.G2	ATGCATCGATGAAGCCCGCGGCAAAAAGCGATATGTAATG	[456]
N3.G1	GTGCATCGATGAAGCCCGCGGCAAAAAGCGATATGTAATG	[456]
N3.G2	GTGCATCGATGAAGCCCGCGGCAAAAAGCGATATGTAATG	[456]
N4.G1	ATGCATCGATGAAGCCCGCGGCAAAAAGCGATATGTAATG	[456]
N4.G2	ATGCATCGATGAAGCCCGCGGCAAAAAGCGATATGTAATG	[456]
N5.G1	ATGCATCGATGAAGCCCGCGGCAAAAAGCGATATGTAATG	[456]
N5.G2	ATGCATCGATGAAGCCCGCGGCAAAAAGCGATATGTAATG	[456]
H1	CCACGCCGATGAAGAGCGCGGCGAAACGCGATATGTCGTG	[456]
N. canariensis (FJ475124.1)	ATGCATCGATGAAGCCCGCGGCAAAAAGCGATATGTAATG	[456]
N. neodobsoni (AM157661.1)	GTGCATCGATGAAGCCCGCGGCAAAAAGCGATATGTAATG	[456]
Hartmannella vermiformis (GU001158.1:186-863)	CCACGCCGATGAAGAGCGCGGCGAAACGCGATATGTCGTG	[456]
N. gruberi (AB298288.1)	ATGCATCGATGAAGCCCGCGGCAAAAAGCGATATGTAATG	[456]
N. gruberi (MG699123.1)	ATGCATCGATGAAGCCCGCGGCAAAAAGCGATATGTAATG	[456]
N. dobsoni (KU380484.1)	GTGCATCGATGAAGCCCGCGGCAAAAAGCGATATGTAATG	[456]
N. sp. 4542 (JQ271648.1)	GTGCATCGATGAAGCCCGCGGCAAAAAGCGATATGTAATG	[456]
N. australiensis (AB128052.1)	ATGCATCGATGAAGCCCGCGGCAAAAAGCGATATGTAATG	[456]
N. australiensis (AB128053.1)	ATGCATCGATGAAGCCCGCGGCAAAAAGCGATATGTAATG	[456]
Acanthamoeba castellanii (KT185626.1:2448-357	TCGCGAGGATGAAGAACGCAGCGAAATGCGATACGTAGTG	[456]
Vermamoeba vermiformis (KT185625.1:2842-350	CCACGCCGATGAAGAGCGCGGCGAAACGCGATATGTCGTG	[456]
Vahlkampfia avara (LC191903.1)	TTGTGACGATGAAGAACGTAGCAAGTTGCGAAAAGTAATG	[456]
Vahlkampfia ciguana (AJ973126.1)	TTGTGACGATGAAGAACGTAGCAAGTTGCGAAAAGTAATG	[456]
Hartmannella sp. (HE617186.1)	CCACGCCGATGAAGAGCGCGGCGAAACGCGATATGTCGTG	[456]

N. australiensis (AJ132034.1)	AGATTCGTTAGCCTCGCGATTCATCAAATTGGTGAACACA	[497]
N. gruberi (AJ132024.1)	AGATTCGTTAGCCTCGCGATTCATCAAATTGGTGAACACA	[497]
N. gruberi (AJ132031.1)	AGATTCGTTAGCCTCGCGATTCATCAAATTGGTGAACACA	[497]
N. philippinensis (AY033618.1)	AGATTCGTTAGCCTCGCGATTCATCAAATTGGTGAACACA	[497]
N. dobsoni (AJ566627.1)	AGATTCGTTAGCCTCGCGATTCATCAAATTGGTGAACACA	[497]
N. paradobsoni (AM157664.1)	AGATTCGTTAGCCTCGCGATTCATCAAATTGGTGAACACA	[497]
N1	AGATTCGTTAGCCTCGCGATTCATCAAATTGGTGAACACA	[497]
N2.G1	AGATTCGTTAGCCTCGCGATTCATCAAATTGGTGAACACA	[497]
N2.G2	AGATTCGTTAGCCTCGCGATTCATCAAATTGGTGAACACA	[497]
N3.G1	AGATTCGTTAGCCTCGCGATTCATCAAATTGGTGAACACA	[497]
N3.G2	AGATTCGTTAGCCTCGCGATTCATCAAATTGGTGAACACA	[497]
N4.G1	AGATTCGTTAGCCTCGCGATTCATCAAATTGGTGAACACA	[497]
N4.G2	AGATTCGTTAGCCTCGCGATTCATCAAATTGGTGAACACA	[497]
N5.G1	AGATTCGTTAGCCTCGCGATTCATCAAATTGGTGAACACA	[497]
N5.G2	AGATTCGTTAGCCTCGCGATTCATCAAATTGGTGAACACA	[497]
H1	CGAATCGCAGAATTCGCGAATCACGCAATCCTCGAACGCA	[497]
N. canariensis (FJ475124.1)	AGATTCGTTAGCCTCGCGATTCATCAAATTGGTGAACACA	[497]
N. neodobsoni (AM157661.1)	AGATTCGTTAGCCTCGCGATTCATCAAATTGGTGAACACA	[497]
Hartmannella vermiformis (GU001158.1:186-863)	CGAATCGCAGAATTCGCGAATCACGCAATCCTCGAACGCA	[497]
N. gruberi (AB298288.1)	AGATTCGTTAGCCTCGCGATTCATCAAATTGGTGAACACA	[497]
N. gruberi (MG699123.1)	AGATTCGTTAGCCTCGCGATTCATCAAATTGGTGAACACA	[497]
N. dobsoni (KU380484.1)	AGATTCGTTAGCCTCGCGATTCATCAAATTGGTGAACACA	[497]
N. sp. 4542 (JQ271648.1)	AGATTCGTTAGCCTCGCGATTCATCAAATTGGTGAACACA	[497]
N. australiensis (AB128052.1)	AGATTCGTTAGCCTCGCGATTCATCAAATTGGTGAACACA	[497]
N. australiensis (AB128053.1)	AGATTCGTTAGCCTCGCGATTCATCAAATTGGTGAACACA	[497]
Acanthamoeba castellanii (KT185626.1:2448-357	TGAATCGCAGGGATCGTGAATCATCGAATCTTTGAACGCA	[497]
Vermamoeba vermiformis (KT185625.1:2842-350	CGAATCGCAGAATTCGCGAATCACGCAATCCTCGAACGCA	[497]
Vahlkampfia avara (LC191903.1)	TGACATGCAAAACTCGTGAATCATCGAATTTTCGAACATA	[497]
Vahlkampfia ciguana (AJ973126.1)	TGACATGCAAAACTCGTGAATCATCAAATTTTCGAACATA	[497]
Hartmannella sp. (HE617186.1)	CGAATCGCAGAATTCGCGAATCACGCAATCCTCGAACGCA	[497]

ITS2

```
N. australiensis (AJ132034.1)                TACTGGACCTCTTCGGAGGTACTTGCGTTAGAGTGCTAGT [551]
N. gruberi (AJ132024.1)                      TACTGGACCTCTTCGGAGGTACTTGCGTTAGAGTGCTAGT [551]
N. gruberi (AJ132031.1)                      TACTGGACCTCTTCGGAGGTACTTGCGTTAGAGTGCTAGT [551]
N. philippinensis (AY033618.1)               TACTGGACCTCTTCGGAGGTACTTGCGTTAGAGTGCTAGT [551]
N. dobsoni (AJ566627.1)                      TACTGGACCTCTTCGGAGGTACTTGCGTTAGAGTGCTAGT [551]
N. paradobsoni (AM157664.1)                  TACTGGACCTCTTCGGAGGTACTTGCGTTAGAGTGCTAGT [551]
N1                                           TACTGGACCTCTTCGGAGGTACTTGCGTTAGAGTGCTAGT [551]
N2.G1                                        TACTGGACCTCTTCGGAGGTACTTGCGTTAGAGTGCTAGT [551]
N2.G2                                        TACTGGACCTCTTCGGAGGTACTTGCGTTAGAGTGCTAGT [551]
N3.G1                                        TACTGGACCTCTTCGGAGGTACTTGCGTTAGAGTGCTAGT [551]
N3.G2                                        TACTGGACCTCTTCGGAGGTACTTGCGTTAGAGTGCTAGT [551]
N4.G1                                        TACTGGACCTCTTCGGAGGTACTTGCGTTAGAGTGCTAGT [551]
N4.G2                                        TACTGGACCTCTTCGGAGGTACTTGCGTTAGAGTGCTAGT [551]
N5.G1                                        TACTGGACCTCTTCGGAGGTACTTGCGTTAGAGTGCTAGT [551]
N5.G2                                        TACTGGACCTCTTCGGAGGTACTTGCGTTAGAGTGCTAGT [551]
H1                                           TCCGGCGCCTTCCCGGGAGCATGCCCGTCTGAGCGTCGTT [551]
N. canariensis (FJ475124.1)                  TACTGGACCTCTTCGGAGGTACTTGCGTTAGAGTGCTAGT [551]
N. neodobsoni (AM157661.1)                   TACTGGACCTCTTCGGAGGTACTTGCGTTAGAGTGCTAGT [551]
Hartmannella vermiformis (GU001158.1:186-863) TCCGGCGCCTTCCCGGGAGCATGCCCGTCTGAGCGCCGTT [551]
N. gruberi (AB298288.1)                      TACTGGACCTCTTCGGAGGTACTTGCGTTAGAGTGCTAGT [551]
N. gruberi (MG699123.1)                      TACTGGACCTCTTCGGAGGTACTTGCGTTAGAGTGCTAGT [551]
N. dobsoni (KU380484.1)                      TACTGGACCTCTTCGGAGGTACTTGCGTTAGAGTGCTAGT [551]
N. sp. 4542 (JQ271648.1)                     TACTGGACCTCTTCGGAGGTACTTGCGTTAGAGTGCTAGT [551]
N. australiensis (AB128052.1)                TACTGGACCTCTTCGGAGGTACTTGCGTTAGAGTGCTAGT [551]
N. australiensis (AB128053.1)                TACTGGACCTCTTCGGAGGTACTTGCGTTAGAGTGCTAGT [551]
Acanthamoeba castellanii (KT185626.1:2448-357 AGTTGCGCCCCCCCGGGAGCACGTTCGCTTGAGTGCCGCT [551]
Vermamoeba vermiformis (KT185625.1:2842-350  TCCGGCGCCTTCCCGGGAGCATGCCCGTCTGAGCGTCGTT [551]
Vahlkampfia avara (LC191903.1)               TCCAATGCGAATTGCGAAGCGCTTACTTGTGAGTGCCAGT [551]
Vahlkampfia ciguana (AJ973126.1)             GACAATGCGAGCTGAGAAGCGCTTGCTCGTGAGTGCCAGT [551]
Hartmannella sp. (HE617186.1)                TCCGGCGCCTTCCCGGGAGCATGCCCGTCTGAGCGTCGTT [551]

N. australiensis (AJ132034.1)                TTTATCAATTGATCTGGTAAAGGTGTTTGAATCGTTAGTT [618]
N. gruberi (AJ132024.1)                      TTTATCAATTGATCTGGTAAAGGTGTTTGAATCATTA?TT [618]
N. gruberi (AJ132031.1)                      TTTATCAATTGATCTGGTAAAGGTGTTTGAATCATTAATT [618]
N. philippinensis (AY033618.1)               TTTATCAATTGATCTGGTAAAGGTGTTTGAATCATTAGTT [618]
N. dobsoni (AJ566627.1)                      TTTATCAATTGAGCCGATAAAGGTGTTTTAATCTTTAGTT [618]
N. paradobsoni (AM157664.1)                  TTTATCAATTGAGCCGATAAAGGTGTTTTAATCTTTAGTT [618]
N1                                           TTTATCAATTGATCTGGTAAAGGTGTTTGAATCATTAGTT [618]
N2.G1                                        TTTATCAATTGATCTGGTAAAGGTGTTTGAATCGTTAGTT [618]
N2.G2                                        TTTATCAATTGATCTGGTAAAGGTGTTTGAATCATTAGTT [618]
N3.G1                                        TTTATCAATTGAGCCGATAAAGGTGGTTTAATCTTTAGTT [618]
N3.G2                                        TTTATCAATTGAGCCGATAAAGGTGTTTTAATCTTTAGTT [618]
N4.G1                                        TTTATCAATTGATCTGGTAAAGGTGTTTGAATCGTTAGTT [618]
N4.G2                                        TTTATCAATTGATCTGGTAAAGGTGTTTGAATCGTTAGTT [618]
N5.G1                                        TTTATCAATTGATCTGGTAAAGGTGTTTGAATCATTAATT [618]
N5.G2                                        TTTATCAATTGATCTGGTAAAGGTGTTTGAATCATTAGTT [618]
H1                                           CCCATCCCTCAGACGTCCGAAGGTAGGGTGATCGGGACGT [618]
N. canariensis (FJ475124.1)                  TTTATCAATTGATCTGGTAAAGGTGTTTGAATCATTAGTT [618]
N. neodobsoni (AM157661.1)                   TTTATCAATTGAGCCGATAAAGGTGTTTTAATCTTTAGTT [618]
Hartmannella vermiformis (GU001158.1:186-863) CCAATCCCAACGACGATTAAAGGATGGGCGATCGGGACAG [618]
N. gruberi (AB298288.1)                      TTTATCAATTGATCTGGTAAAGGTGTTTGAATCATTAATT [618]
N. gruberi (MG699123.1)                      TTTATCAATTGATCTGGTAAAGGTGTTTGAATCATTAATT [618]
N. dobsoni (KU380484.1)                      TTTATCAATTGAGCCGATAAAGGTGTTTTAATCTTTAGTT [618]
N. sp. 4542 (JQ271648.1)                     TTTATCAATTGAGCCGATAAAGGTGTTTTAATCTTTAGTT [618]
N. australiensis (AB128052.1)                TTTATCAATTGATCTGGTAAAGGTGTTTGAATCGTTAGTT [618]
N. australiensis (AB128053.1)                TTTATCAATTGATCTGGTAAAGGTGTTTGAATCGTTAGTT [618]
Acanthamoeba castellanii (KT185626.1:2448-357 TCTATCCATTAAGCTCATTCCGGTGGTGGAATTGGGGGGC [618]
Vermamoeba vermiformis (KT185625.1:2842-350  CCAATCCCAAAGACGATCGAAGGATGGGTGATCGGGACAG [618]
Vahlkampfia avara (LC191903.1)               TTTTCTTACTGGACTGAGAAACGAAGCCGGGTTTCCGTTT [618]
Vahlkampfia ciguana (AJ973126.1)             TATCCTTACTGGACTGAGATATGCTGATGCACAGTCATGT [618]
Hartmannella sp. (HE617186.1)                CCCATCCCTCAGACGTCCGAAGGTAGGGTGATCGGGACGT [618]

N. australiensis (AJ132034.1)                TTACCCCTAGCCGGGGAGCCGGATTCCTGAGAATTCGATT [670]
N. gruberi (AJ132024.1)                      TTACCCCTAGCCGGGTAGCCGGATTCCTGAGAATTCGATT [670]
N. gruberi (AJ132031.1)                      TTACCCCTAGCCGGGTAGCCGGATTCCTGAGAATTCGATT [670]
N. philippinensis (AY033618.1)               TTACCCCTAGCCGGGTAGCCGGATTCCTGAGAATTCGATT [670]
N. dobsoni (AJ566627.1)                      TTACCCCGAGTCGGGAAGCCGGTTTCTTGAGAATCCGATT [670]
N. paradobsoni (AM157664.1)                  TTACCCCGAGTCGGGAAGCCGGTTTCTTGAGAATCCGATT [670]
N1                                           TTACCCCTAGCCGGGTAGCCGGATTCTTGAGAATTCGATT [670]
N2.G1                                        TTACCCCTAGCCGGGTAGCCGGATTCCTGAGAATTCGATT [670]
N2.G2                                        TTACCCCTAGCCGGGTAGCCGGATTCCTGAGAATTCGATT [670]
N3.G1                                        TTACCCCGAGTCGGGAAGCCGGTTTCTTGAGAATCCGATT [670]
N3.G2                                        TTACCCCGAGTCGGGAAGCCGGTTTCTTGAGAATCCGATT [670]
N4.G1                                        TTACCCCTAGCCGGGGAGCCGGATTCCTGAGAATTCGATT [670]
N4.G2                                        TTACCCCTAGCCGGGGAGCCGGATTCCTGAGAATTCGATT [670]
N5.G1                                        TTACCCCTAGCCGGGTAGCCGGATTCCTGAGAATTCGATT [670]
N5.G2                                        TTACCCCTAGCCGGGTAGCCGGATTCCTGAGAATTCGATT [670]
H1                                           CGATGCAGTTCCTGGGAACAGGCGCACCTACTACCCCGTC [670]
N. canariensis (FJ475124.1)                  TTACCCCTAGCCGGGTAGCCGGATTCTTGAGAATTCGATT [670]
N. neodobsoni (AM157661.1)                   TTACCCCGAGTCGGGAAGCCGGTTTCTTGAGAATCCGATT [670]
Hartmannella vermiformis (GU001158.1:186-863) AAATGCAGTTTCTGGGAACAGGCGCATCGAAAGCCCCATC [670]
N. gruberi (AB298288.1)                      TTACCCCTAGCCGGGTAGCCGGATTCCTGAGAATTCGATT [670]
N. gruberi (MG699123.1)                      TTACCCCTAGCCGGGTAGCCGGATTCCTGAGAATTCGATT [670]
N. dobsoni (KU380484.1)                      TTACCCCGAGTCGGGAAGCCGGTTTCTTGAGAATCCGATT [670]
N. sp. 4542 (JQ271648.1)                     TTACCCCGAGTCGGGAAGCCGGTTTCTTGAGAATCCGATT [670]
N. australiensis (AB128052.1)                TTACCCCTAGCCGGGGAGCCGGATTCCTGAGAATTCGATT [670]
N. australiensis (AB128053.1)                TTACCCCTAGCCGGGGAGCCGGATTCCTGAGAATTCGATT [670]
Acanthamoeba castellanii (KT185626.1:2448-357 TTACACCGCGGCGGGTGGCTGGCCTCTTGTGTATGGTATT [670]
Vermamoeba vermiformis (KT185625.1:2842-350  CGATGCAGTTTCTGGGAACAGGCGCACCGATTGCCCCATC [670]
Vahlkampfia avara (LC191903.1)               TCTCTCCATAGTAAGAAAGTGGACTTATGACAGTTCTTTT [670]
Vahlkampfia ciguana (AJ973126.1)             TCTCTCCATAGTAAGAAAGTGAACTTATGACAGTTCTTTT [670]
Hartmannella sp. (HE617186.1)                CGATGCAGTTCCTGGGAACAGGCGCACCTACTACCCCGTC [670]
```

LSU

N. australiensis (AJ132034.1)	T T C C G T C T T T G A C T G G T C A A T C A T T T T A T T	
N. gruberi (AJ132024.1)	T T C C T G T T T T G A C T A G T C A A T C T T T ? ? A T T	[782]
N. gruberi (AJ132031.1)	T T C C T G T T T T G A C T A G T C A A T C T T T T T A T T	[782]
N. philippinensis (AY033618.1)	T T C C G G T T T T G A C T A G T C A A T C C T T G T A T T	[782]
N. dobsoni (AJ566627.1)	T C C T G A G T T G A A C T G G A T T A C G C T C T G A T C	[782]
N. paradobsoni (AM157664.1)	T C C T G A G T T G A A C T G G A T T A C G C T C T G A T C	[782]
N1	T T C C G A A T T T G A C T A G T C A A T C C T T T T A T T	[782]
N2.G1	T T C C G G T T T T G A C T A G T C A A T C C T T G T A T T	[782]
N2.G2	T T C C G G T T T T G A C T A G T C A A T C C T T G T A T T	[782]
N3.G1	T C C T G A G T T G A A C T G G A T T A C G C T C T G A T C	[782]
N3.G2	T C C T G A G T T G A A C T G G A T T A C G C T C T G A T C	[782]
N4.G1	T T C C G T C T T T G A C T G G T C A A T C A T T T T A T T	[782]
N4.G2	T T C C G T C T T T G A C T G G T C A A T C A T T T T A T T	[782]
N5.G1	T T C C T G T T T T G A C T A G T C A A T C T T T T T A T T	[782]
N5.G2	T T C C G G T T T T G A C T A G T C A A T C T T T T T A T T	[782]
H1	G A C T G A G G C G G C C C G A T C C A T C C T C C G G T C	[782]
N. canariensis (FJ475124.1)	T T C C G A A T T T G A C T A G T C A A T C C T T T T A T T	[782]
N. neodobsoni (AM157661.1)	T C C T G A G T T G A A C T G G A T T A C G C T C T A A T C	[782]
Hartmannella vermiformis (GU001158.1:186-863)	G A C G G A G G T G C C C T A A T C C A T T C T C T C G T C	[782]
N. gruberi (AB298288.1)	T T C C T G T T T T G A C T A G T C A A T C T T T T T A T T	[782]
N. gruberi (MG699123.1)	T T C C T G T T T T G A C T A G T C A A T C T T T T T A T T	[782]
N. dobsoni (KU380484.1)	T C C T G A G T T G A A C T G G A T T A C G C T C T G A T C	[782]
N. sp. 4542 (JQ271648.1)	T C C T G A G T T G A A C T G G A T T A C G C T C T G A T C	[782]
N. australiensis (AB128052.1)	T T C C G T C T T T G A C T G G T C A A T C A T T T T A T T	[782]
N. australiensis (AB128053.1)	T T C C G T C T T T G A C T G G T C A A T C A T T T T A T T	[782]
Acanthamoeba castellanii (KT185626.1:2448-357	T G C C A A C A T T G G T C G G T G T G T T G G G G T T	[782]
Vermamoeba vermiformis (KT185625.1:2842-350	G A C G G A G G T G C C C T G A A C C A T T C T C T C G T C	[782]
Vahlkampfia avara (LC191903.1)	T G T A A A C T C T G T G A G G G T T A C C C T T T A A G C	[782]
Vahlkampfia ciguana (AJ973126.1)	C A T A A A C T C T G C G A G G G T T A C C C T T T A A G C	[782]
Hartmannella sp. (HE617186.1)	G A C T G A G G C G G C C C G A T C C A T C C T C C G G T C	[782]
